# Cost-Effective and High-Throughput WSPRi Sensing System Based on Multi-Monochromatic LEDs and Adaptive Second-Order Fitting Algorithm

**DOI:** 10.3390/s26010036

**Published:** 2025-12-20

**Authors:** Chenglong Guo, Jiacong Xiao, Jianchun Zeng, Youjun Zeng, Yi Liu

**Affiliations:** 1 School of Physics and Optoelectronic Engineering, Guangdong University of Technology, Guangzhou 510006, China; gdutguo2025@163.com; 2 Guangdong Provincial Key Laboratory of Sensing Physics and System Integration Applications, Guangdong University of Technology, Guangzhou 510006, China; 3 The First Affiliated Hospital, Guangzhou University of Traditional Chinese Medicine, Guangzhou 510400, Chinazjccsr2005@126.com (J.Z.)

**Keywords:** surface plasmon resonance, biosensing, bioimaging, biophotoncis, plasmonics

## Abstract

Surface Plasmon Resonance imaging (SPRi) is a powerful label-free technique for high-throughput biochemical analysis. Wavelength modulation is particularly suitable for SPRi due to its wide dynamic range and robustness to fabrication tolerances. However, conventional systems relying on tunable filters (e.g., AOTF, LCTF) suffer from high cost, complexity, and limited temporal resolution. To overcome these drawbacks, we developed a rapid wavelength-modulation SPRi system using a multi-LED source and an adaptive second-order fitting (ASF) algorithm. The system covers the 730–805 nm spectrum with five LEDs. The ASF algorithm first performs a coarse full-spectrum scan to locate the resonance wavelength, then dynamically selects an optimal three-LED subset for fast second-order fitting, enabling accurate reconstruction of resonance wavelength without mechanical scanning. This approach significantly reduces cost and complexity while achieving a scanning cycle of 105 ms, RI resolution of 5.54 × 10^−6^ RIU, dynamic range of 0.0241 RIU, and excellent multi-channel consistency. The system has been successfully applied to monitor multi-channel antibody–antigen interactions in real time. Furthermore, it was used to detect cartilage oligomeric matrix protein (COMP) in synovial fluid, where an elevated concentration in an osteoarthritis sample versus a control aligned with its role as a cartilage catabolism marker. This work validates a practical and reliable platform for early diagnosis of osteoarthritis.

## 1. Introduction

Surface plasmon resonance (SPR) sensing technology has become a prominent tool in biochemical analysis and clinical screening and diagnosis, owing to its high sensitivity and label-free operation [1,2]. The integration of SPR with imaging techniques, referred to as SPR imaging (SPRi), further enables parallel detection of multiple samples, significantly enhancing analytical throughput [3,4,5]. Current SPR sensing systems are primarily based on four modulation schemes: intensity, angle, phase, and wavelength modulation [6]. Among these, intensity-modulated SPR operates at a fixed incident angle and wavelength by monitoring variations in reflected light intensity [7]. This approach offers the advantages of a simple optical setup and ease of implementation, making it the most widely used modality [8,9,10]. However, it is susceptible to light source fluctuations and external noise, generally achieving a refractive index resolution (RIR) on the order of 10^−5^ RIU and a dynamic range of approximately 10^−3^ RIU [11,12]. Moreover, fabrication tolerances in the metal layer thickness (typically within ~5 nm) of the sensing chip, combined with the constrained dynamic range, hinder consistent multi-site detection, thereby limiting its applicability in spatially resolved sensing [13]. In contrast, angle-modulated SPR provides improved sensitivity and a broader dynamic range, with an RIR of ~10^−6^ RIU and a dynamic range of ~10^−2^ RIU [14,15]. This method employs a fixed wavelength while scanning the incident angle—or analyzing the angular reflectance spectrum—to identify the resonance angle, which shifts in response to RI changes. However, in prism-coupled configurations, varying the incident angle can induce image distortion, preventing true in situ multi-point monitoring. Additionally, laser sources commonly used in such systems to enhance sensitivity are prone to speckle noise, which degrades image quality and restricts their utility in imaging applications [16]. Phase-modulated SPR operates by fixing both the angle and wavelength while modulating the phase of the incident light; the phase difference between the incident and reflected beams is then demodulated to reflect sample changes [17,18]. This method exhibits ultra-high sensitivity but is constrained by a narrow dynamic range (~10^−4^ RIU) and is highly sensitive to variations in metal layer thickness, resulting in poor site-to-site consistency [19].

In this context, wavelength modulation stands out as the most suitable technique for SPR imaging, primarily due to its unique combination of imaging compatibility and operational robustness [20]. By operating at a fixed incident angle, it completely avoids the image distortion associated with mechanical angle scanning, enabling true in situ and parallel monitoring across the entire sensor field. Furthermore, its exceptionally broad dynamic range provides high tolerance to variations in metal layer thickness, ensuring consistent sensor response and signal uniformity across all imaging channels [21]. Crucially, the ability to flexibly select optimal excitation wavelengths for different analytes offers a unique degree of freedom for optimizing contrast and sensitivity in imaging experiments [22,23]. These attributes collectively make wavelength modulation the best choice for SPRi sensors.

Wavelength-modulated SPR sensing was initially demonstrated by Yuk et al., who employed a spectrometer to scan the sensing surface, acquiring SPR spectral curves and corresponding resonance wavelength (RW) for multi-point sensing [24,25]. However, this RW imaging approach is inherently slow, resulting in the loss of dynamic biological information and failing to monitor fast reaction kinetics. To accelerate imaging, subsequent methods introduced wavelength-scanning illumination and area-scanning modes for two-dimensional sensing surface imaging. These typically utilize continuous beam splitters—such as monochromators, liquid crystal tunable filter (LCTF) [26,27], or acousto-optic tunable filter (AOTF)—to disperse broadband light [28,29], combined with array detectors (e.g., CMOS or CCD cameras) to image the sensing surface. While such array-based imaging significantly improves acquisition speed, it still relies on sequential wavelength scanning. Our team previously developed several high-speed RW imaging schemes that incorporate continuous beam splitters (e.g., LCTF, AOTF) and RW-tracking algorithms, reducing the number of spectral points required per cycle and achieving imaging speeds of up to 0.2 s/frame [28,30]. Nevertheless, all these approaches inevitably depend on tunable filters, which are not only costly but also increase system complexity and compromise long-term stability.

In this paper, we present a novel, cost-effective, and high-speed wavelength-modulated SPR imaging (WSPRi) system that overcomes the limitations of traditional tunable-filter-based approaches. The core of our system is an adaptive second-order fitting (ASF) algorithm, which works in conjunction with a multi-LED illumination source to achieve rapid RW reconstruction without mechanical scanning. We detail the working principle and implementation of the ASF algorithm and systematically characterize the system’s performance in terms of sensitivity, dynamic range, and multi-channel consistency. Based on this system, we have achieved parallel and real-time monitoring of multi-channel antigen–antibody interactions. Furthermore, the practical utility of the system is demonstrated through the detection of cartilage oligomeric matrix protein (COMP) in human synovial fluid, showcasing its potential for clinically relevant biomarker analysis and paving the way for precise molecular-level monitoring in applications such as osteoarthritis management.

## 2. Setup and Materials

### 2.1. Optical Configuration

**Figure 1:**
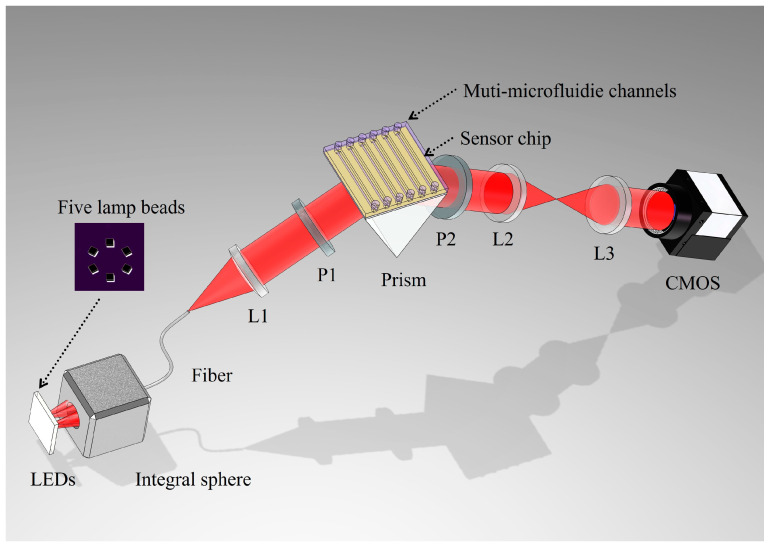
The schematic diagram of system optical path, L1–3 lens, P1–2 polarizer.

The optical path of the system is illustrated in Figure 1, comprising three main sections: the incident light path, the sensing module, and the reflected light path. The excitation light source in the incident path consists of five monochromatic LEDs with center wavelengths of 730, 740, 576, 777, and 805 nm. The light from these LEDs is coupled into a multimode fiber via an integrating sphere. The integrating sphere is a RK-36T, Ruike Photoelectric technology Co., Led. Each LED provides approximately 2 mW optical power. After coupling and collimation, the total power incident on the prism is 0.2–0.3 mW, with 0.1 mW reaching the CMOS sensor after reflection and imaging optics (without SPR). Upon emission from the fiber, the light is collimated by lens L1, passes through polarizer P1 (oriented in the -p direction), and then enters the sensing module. This module, which includes a prism, a sensing chip, and a flow cell, facilitates the coupling of incident light via the prism to excite surface plasmon resonance (SPR) at the sensing chip-sample interface. The prism is an equilateral triangle (side length 18 mm) made of SF11 glass (*n* = 1.785 at 633 nm). The sensor chip is gently pressed against the prism using a spring-loaded clamp, with a drop of index-matching oil (*n* = 1.780 RIU) applied to eliminate air gaps. The reflected light subsequently passes through polarizer P2 (aligned with the -p polarization direction) and the imaging lens group (L2 and L3), before being captured by a CMOS sensor. During operation, each monochromatic LED is illuminated sequentially, and the CMOS records an image of the entire sensing surface for each wavelength. A key feature of this system is the ability to select any region of interest (ROI) on the captured sensing image for subsequent detailed analysis. The CMOS camera is a DMK 33GP031, Imaging Source, Bremen, Germany. The system is controlled by home-made Labview software (2019,32-bit). The sensing area is defined by the CMOS field of view (≈10 mm × 7.5 mm). The PDMS microfluidic chip used contains six parallel channels, each 1 mm wide × 10 mm long.

The selection of the 730–805 nm spectral range was based on several considerations: (1) longer wavelengths can enhance system sensitivity; (2) this range aligns with the high-efficiency operational band of optical components (lenses, prism, CMOS), maximizing light throughput and signal-to-noise ratio; (3) commercial availability of high-power (>10 mW) monochromatic LEDs in this region ensures sufficient excitation intensity. The use of five LEDs represents a balance between dynamic range, system complexity, and cost. While additional LEDs could extend the dynamic range, they would increase cost, and complexity, with diminishing returns for typical biosensing applications.

### 2.2. Materials and Chemicals

Materials: The sensor chip in the system is based on 1 mm thick of glass (Length: 18 mm, width: 18 mm) as the substrate, with a 48 nm-thick of gold layer being deposited on the substrate through evaporation, and 2 nm of chromium layer spacing between the glass substrate and the gold coating to increase the fixation effect. The prism is equilateral triangle with side length of 18 mm made by SF11 (1.785 RIU).

Chemicals: The goat anti-rabbit IgG, rabbit IgG, bovine serum albumin (BSA), anti-COMP polyclonal antibody, and phosphate-buffered saline (PBS) were obtained from Solarbio Science & Technology Co., Ltd. (Beijing, China). Refractive index matching oil (1.780 RIU) was bought from Cargille Laboratories, Inc. (Cedar Grove, NJ, USA). NaCl was obtained from Aladdin (Shanghai, China).

Clinical samples: The synovial fluid samples were offered by the first affiliated hospital, Guangzhou University of Traditional Chinese Medicine.

## 3. Theory and Algorithm

### 3.1. SPR Theory

According to the Kretschmann configuration, surface plasmons (SPs) are excited at the interface between the metal layer and the dielectric medium. When light of an appropriate wavelength and incident angle illuminates the sensing chip, a sharp drop in reflectivity occurs due to resonance between the SPs and transverse magnetic (TM)-polarized light, resulting in the characteristic SPR dip. The resonance condition is influenced by several factors, including the incident angle, wavelength, sensing layer material, and the RI of the prism.

The surface plasmon wave (SPW) propagates along the metal–dielectric interface, with its propagation constant $k_{SP}$ given by [31]:

(1) $$k_{SP}=\frac{2\pi }{\lambda }\sqrt{\frac{\varepsilon_{m}\left(\lambda \right)\cdot \varepsilon_{d}\left(\lambda \right)}{\varepsilon_{m}\left(\lambda \right)+\varepsilon_{d}\left(\lambda \right)}}$$

where $\varepsilon_{m}(\lambda )$ and $\varepsilon_{d}(\lambda )$ represent the wavelength-dependent dielectric constants of the metal and the dielectric medium, respectively.

When incident light reaches the prism surface, the component of its wave vector along the interface (*x*-axis) is expressed as [32]:

(2) $$k_{x}=\frac{2\pi }{\lambda }n_{p}\left(\lambda \right)\sin\theta$$

Here, $n_{p}(\lambda )$ denotes the RI of the prism, and θ is the incident angle measured from the normal to the prism surface. Resonance occurs when $k_{SP}\approx k_{x}$, leading to a pronounced attenuation in the reflected light intensity, which manifests as the SPR dip. The corresponding wavelength at this minimum is defined as the RW.

### 3.2. Adaptive Second-Order Fitting Algorithm

The SPR spectral curve is inherently nonlinear and exhibits high-order characteristics. For example, a typical spectrum with a 100 nm bandwidth can generally be modeled by a seventh-order polynomial. However, within the critical region surrounding the RW—spanning approximately 35 nm—the curve can be accurately approximated by a second-order function. By acquiring three data points within this narrow band and performing a second-order fit, the local spectral profile can be effectively reconstructed. While this method enhances the system’s scanning speed by minimizing the number of sampling points, the associated dynamic range for this narrow bandwidth is limited to 10^−3^ RIU. To simultaneously achieve high-speed detection and a large dynamic range, we propose an adaptive second-order fitting (ASF) algorithm.

**Figure 2:**
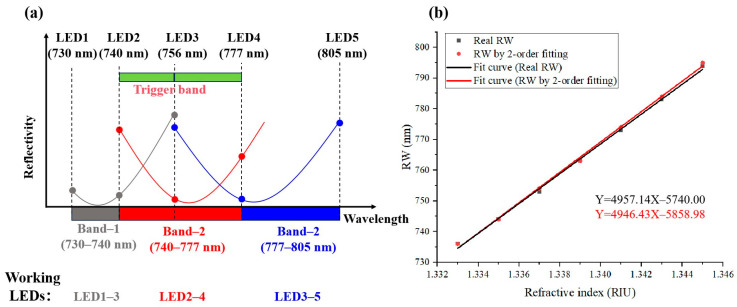
Adaptive second-order fitting algorithm: (**a**) Schematic diagram of the principle of ASF algorithm; (**b**) The linear fitting of the RW via ASF algorithm and the real RW with RI.

Figure 2a presents a schematic of the proposed adaptive algorithm. The five LEDs are designated as LED1 to LED5 in ascending order of their central wavelengths. The operational spectrum (730–805 nm) is partitioned into three sub-bands: Band1 (730–740 nm), Band2 (740–777 nm), and Band3 (777–805 nm). In the initial phase, all five LEDs are activated sequentially to sample five wavelengths across the full spectrum. A fourth-order polynomial fit is applied to these data points to estimate the initial RW (denoted as the starting λ). Depending on the band in which the initial wavelength is located, the system operates as follows:

Band1: LEDs 1–3 are activated, sampling three specific wavelengths (gray circles) to generate the second-order fitted curve (gray);

Band2: LEDs 2–4 are used, sampling the wavelengths marked by red circles to produce the red fitted curve;

Band3: LEDs 3–5 are employed, with the sampled blue dots yielding the blue fitted curve.

The system is typically initialized by adjusting the incident angle to position the RW within Band1 (730–740 nm). As the sample’s RI increases—such as during biomolecular binding events—the RW undergoes a red-shift. The algorithm adapts to this shift by dynamically switching the active LED set and its associated fitting band during scanning:

When the RW ≥ 740 nm, the system switches from LED1–3 to LED2–4, shifting the fitting band to 740–777 nm;

With a further increase to ≥756 nm, LED3–5 are activated, and the fitting band transitions to 756–805 nm.

This adaptive strategy enables rapid reconstruction of the SPR curve within the relevant spectral region while preserving a large dynamic range. Crucially, even when only three LEDs are active, the RW can be accurately computed provided it remains within the excitation range of the selected LEDs. RWs between 740 nm and 756 nm can be resolved using either the spectral curve from LED1–3 or LED2–4. Similarly, RWs between 756 nm and 777 nm can be computed using the curve from either LED2–4 or LED3–5. Consequently, the overlapping regions (740–756 nm and 756–777 nm) function as trigger bands, as indicated by the green rectangle in Figure 2a. The intentional spectral overlap between adjacent bands ensures smooth transitions and computational stability, even for samples exhibiting continuous RI gradients. The system also handles abrupt changes in RI seamlessly through its band-switching logic.

Consider a scenario, as illustrated in Figure 2a, where the system is operating in Band1, while the actual resonance has shifted to 783 nm (represented by the solid blue SPR curve):

(1) Using Band1 (LED1–3), the initial calculated RW is 756 nm.
(2) This result triggers a switch to Band2 (LED2–4), which subsequently yields a refined RW of 777 nm.
(3) The system then activates Band3 (LED3–5), finally determining the true RW of 783 nm.

This stepwise convergence to the accurate value is facilitated by the deliberate spectral overlap between bands, ensuring robust tracking even under significant RI changes.

To validate the computational performance of the proposed ASF algorithm, we conducted a numerical simulation with the following parameters: a prism RI of 1.785, a gold-coated sensing chip, an incident angle of 51.2°, an initial ambient RI of 1.333 RIU, and a RI step of 0.002 RIU. The simulation results are presented in Figure 2b, where the true and ASF-computed RWs are indicated by black and red dots, respectively. Linear regression yielded sensitivities of 4957.14 nm/RIU for true RWs and 4946.43 nm/RIU for ASF-computed RWs with RI change. The relative error in sensitivity introduced by the ASF algorithm is only 0.2%, confirming its high accuracy in tracking RW variations.

Compared to existing wavelength-modulation strategies—such as full-spectrum polynomial fitting or tunable-filter-based sequential scanning (e.g., AOTF, LCTF)—the proposed ASF algorithm offers distinct advantages: (1) it eliminates the need for mechanical scanning or expensive tunable optical components; (2) it adaptively selects a minimal three-wavelength subset, enabling both high speed (105 ms/cycle) and wide dynamic range; (3) it maintains low computational overhead through localized second-order fitting. This approach thus provides a cost-effective, robust, and scalable solution for real-time SPR imaging.

## 4. Results and Discussion

### 4.1. System Performance Testing

**Figure 3:**
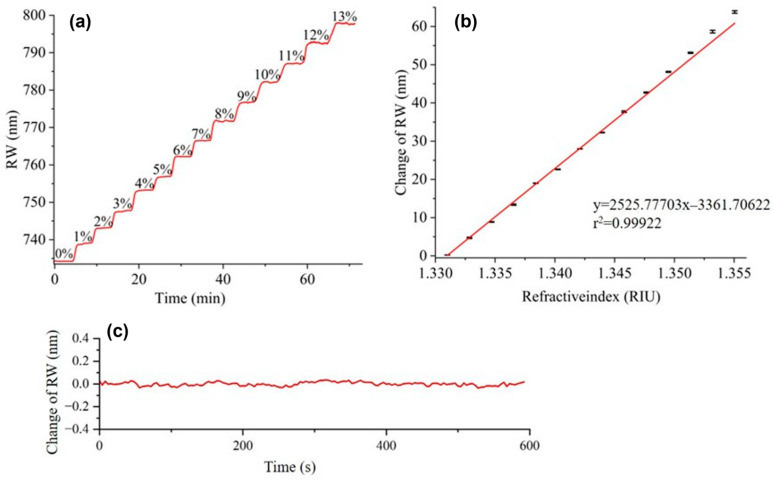
(**a**) The real-time monitoring of RW as NaCl concentration increased from 0% to 13%; (**b**) Linear relationship between RW shift and the RI of NaCl, the error bars visually represent the uncertainty in the data from repeated experiments, which was within 3.8%; (**c**) Examination of the SPR signal shift with pure water for a period of 10 min.

The system’s capability for real-time monitoring of samples with continuously varying refractive index (RI) was validated by tracking the RW shifts in response to different concentrations of NaCl solution. During the experiment, solutions with concentrations ranging from 0% to 13% (in 1% increments) were sequentially injected into the flow cell, while the corresponding RW was recorded in real time. The results are presented in Figure 3a. Furthermore, since the system only requires scanning and imaging at three specific wavelengths per cycle, the minimum time per scanning cycle is 105 ms, calculated as 3 × (2 ms + 33 ms), where 2 ms represents the LED response time and 33 ms the CMOS exposure time. If parallel detection is performed on samples with large RI differences and band1–3 work simultaneously, the minimum time resolution of the system is: 5 × (2 ms + 33 ms) = 175 ms.

Figure 3b shows the RW shift as a function of RI of NaCl solution, revealing a strong linear relationship across the tested range (r^2^ = 0.99922). Since the RI difference between 13% NaCl solution and pure water is 0.0241 RIU, the system’s dynamic range is thereby defined as 0.0241 RIU.

The RIR of the system is another important parameter. According to formula [3], RIR of our system is calculated to be $\sigma_{RI}=5.54\times 10^{-6}\;$ RIU, where $\sigma_{n}$ is the change in refraction index, the $\sigma_{s}$ is the corresponding RW response, and the ${\Delta \sigma }_{SD}=$ 0.014 nm is root mean square (RMS) of noise, which calculated by conducting a 10 min baseline test with the sample of pure water.

(3) $$\sigma_{RI}=\frac{\sigma_{n}}{\sigma_{s}}{\Delta \sigma }_{SD}.$$

To provide a clear perspective on the performance of our system in the context of recent SPRi research, we present a comparative summary of key parameters in Table 1. The table juxtaposes the scanning speed, RIR, dynamic range, and approximate cost/complexity of our LED-based WSPRi system with those of representative wavelength-modulated SPRi platforms reported in the recent literature, including systems employing acousto-optic tunable filters (AOTF), liquid crystal tunable filters (LCTF), and spectrometer-based scanning. This comparison highlights the distinctive advantage of our approach in achieving a favorable balance between high speed, excellent sensitivity, broad dynamic range, and significantly lower cost/complexity.

### 4.2. Detection of Antibody–Antigen Binding

Prior to testing, the sensing chip underwent a three-step pretreatment procedure:

(1) Probe Immobilization: The chip was first rinsed with PBS (0.01 M, pH = 7.3) for 5 min, followed by the introduction of rabbit IgG (100 μg/mL). The antigen was immobilized on the chip surface via physical adsorption. After allowing the signal to stabilize for approximately 10 min, PBS was reintroduced for a 10-min wash.
(2) Blocking: Bovine serum albumin (BSA) at a concentration of 100 μg/mL was then introduced to block any non-specific binding sites.
(3) Final Rinse: A final PBS wash was performed for 10 min to ensure the chip surface was thoroughly cleaned.

**Figure 4:**
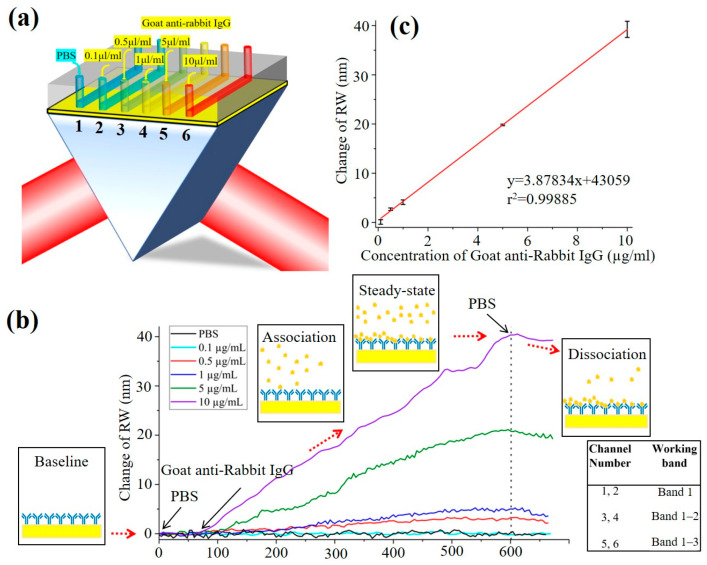
Real time monitoring of antigen–antibody specific binding. (**a**) Schematic diagram of the detection device; (**b**) Real-time measurement of the binding reaction between different concentrations of goat anti-rabbit IgG and rabbit IgG proteins; (**c**) Linear relationship between RW shift and the goat anti-rabbit IgG concentration (*n* = 3), and the error bars visually represent the uncertainty in the data from repeated experiments, which was within 5%.

The experimental setup is depicted in Figure 4a. Initially, PBS was introduced into all six flow channels under continuous SPR signal monitoring. After signal stabilization, channel 1 was maintained with PBS as a negative control, while channels 2–6 were injected with goat anti-rabbit IgG at concentrations of 0.1, 0.5, 1, 5, and 10 μg/mL, respectively. Approximately 8 min post-injection, once all channel signals had stabilized, PBS was reintroduced to rinse the system. Throughout the experiment, the system automatically computed the RW for each channel using the ASF algorithm and dynamically adjusted the working spectral band based on the RW values. Owing to the wide dynamic range of the system, RWs across all channels were effectively monitored.

The real-time RW response curves for each channel are shown in Figure 4b. The initial PBS injection (0–300 s) established a stable baseline. Upon introduction of the test solutions, specific binding of goat anti-rabbit IgG to the surface-immobilized rabbit IgG induced RW shifts, with the response increasing in a concentration-dependent manner. As binding approached saturation at approximately 750 s, all response curves plateaued. Subsequent PBS washing removed unbound and non-specifically adsorbed antibodies, leading to a decrease in RW signals. The net RW shift—calculated as the difference before and after the reaction—primarily reflects the extent of specific binding and shows a positive correlation with the concentration of goat anti-rabbit IgG in the test solution. The corresponding working bands for each channel are summarized in the table embedded in Figure 4b. Figure 4c shows the calibration plot corresponding to the RW shift with increasing protein concentration (0.1–10 μg/mL). The linear correlation coefficient (r^2^) of the data is 0.99885, which exhibits a nearly linear relationship between the RW shift and the protein concentrations ranging from 0.1 to 10 μg/mL.

(4) $$LOD=3\times \frac{\delta_{N}}{S}$$

We also calculated the limit of detection (LOD) of the system based on formula 4 [35]. Where $\delta_{N}$ is the standard deviation of blank, *S* is the slope of the calibration curve. Based on Formula 4, the LOD was calculated as 0.042 μg/mL. This LOD is comparable to or better than those reported in recent label-free SPRi studies for IgG detection [7], underscoring the competitive sensitivity of our system.

### 4.3. Clinical Sample Detection

To demonstrate the practical applicability of our WSPRi sensing system in a clinical-relevant context, we conducted a preliminary detection of COMP in human synovial fluid samples. COMP is a well-established non-collagenous glycoprotein of the cartilage extracellular matrix, and its elevated concentration in synovial fluid is a recognized biomarker reflecting active cartilage catabolism and turnover—a central pathological process in OA [36,37,38]. In this proof-of-concept experiment, synovial fluid from two OA patients and two non-OA donors was analyzed.

**Figure 5:**
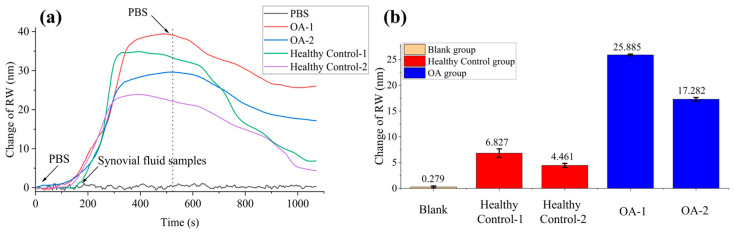
Monitoring of COMP in clinical synovial fluid. (**a**) Real-RW shift response curve; (**b**) The RW shifts before and after the reaction for the two OA groups, two healthy control groups, and one blank group.

Prior to detection, the anti-COMP antibody was immobilized on the chip surface through physical adsorption using a protocol consistent with the rabbit IgG immobilization procedure. The functionalized chip was subsequently blocked with BSA, rinsed with PBS, and integrated into the WSPRi system. Synovial fluid samples were centrifuged at 10,000 rpm for 20 min, and the resulting supernatant was collected as the test analyte. Two OA and two healthy control samples were simultaneously introduced into separate microfluidic channels alongside a PBS reference. Following stabilization of the SPR response, PBS was reintroduced to remove unbound components. Real-time response curves for all channels are presented in Figure 5a.

The observed resonance wavelength (RW) shift is attributed to specific binding between the immobilized anti-COMP antibody and COMP present in the synovial fluid. Results demonstrated significantly greater RW shifts in OA patient samples compared to healthy control, consistent with the expected elevated COMP concentrations in OA synovial fluid [39,40] and aligns with established pathophysiology wherein cartilage degradation in OA leads to release of matrix components into joint fluid [38,41]. Three independent replicate experiments confirmed these observations, with results summarized in Figure 5b. The consistent elevation of COMP levels in OA samples underscores the potential of this biomarker for molecular-level assessment of joint health.

## 5. Conclusions

In this work, we have developed and validated a novel wavelength-modulated SPR imaging (WSPRi) system that addresses the key limitations of cost, complexity, and speed in conventional approaches. The core innovation is the integration of a multi-LED source with an adaptive second-order fitting (ASF) algorithm, enabling rapid and precise resonance wavelength reconstruction without mechanical scanning or expensive tunable filters. The system achieves a scanning speed of 105 ms per cycle, a refractive index resolution of 5.54 × 10^−6^ RIU, and a wide dynamic range of 0.0241 RIU, while maintaining excellent multi-channel consistency—highlighting its advantages of low cost, high speed, and wide dynamic range. The practical utility of the platform was demonstrated through real-time, parallel monitoring of antigen–antibody interactions and the specific detection of the osteoarthritis biomarker COMP in human synovial fluid. The elevated COMP signal in OA samples aligns with established pathophysiology and underscores the system’s potential for high-throughput, molecular-level diagnostics.

In summary, this study presents a cost-effective, high-performance, and versatile WSPRi platform suitable for applications requiring real-time, multi-analyte monitoring, such as biomedical research, drug development, and point-of-care diagnostics. Future work may focus on expanding the dynamic range with additional LEDs, improving sensitivity with lower-noise detectors, and validating the system in larger clinical cohorts for multi-biomarker profiling.

## Figures and Tables

**Table 1:** Comparison of key performance parameters for wavelength-modulated SPRi platforms.

System/Method	Scanning Speed (ms)	RIR (RIU)	Dynamic Range (RIU)	Cost
AOTF-based SPRi [28]	200	$1.17\times 10^{-6}$	0.037	High
LCTF-based SPRi [33]	~4000	$5.87\times 10^{-6}$	0.046	High
Grating-based SPRi [34]	>1000	$8.1\times 10^{-5}$	0.005	Moderate
LEDs and ASF SPRi (This work)	105	$5.5\times 10^{-6}$	0.0241	Low

## Data Availability

No new data were created or analyzed in this study. Data sharing does not apply to this article. We used only publicly available datasets for experimentation.
